# Supplementary material for: Proof of concept of the potential of a machine learning algorithm to extract new information from conventional SARS-CoV-2 rRT-PCR results
Source: Sci Rep. 2023 May 13;13:7786. doi: 10.1038/s41598-023-34882-6 (PMC10182547; doi:10.1038/s41598-023-34882-6)
Supplement: Supplementary file 1 — Supplementary Information. [file 41598_2023_34882_MOESM1_ESM.pdf]

Proof of concept of the potential of a Machine Learning algorithm to extract new information from conventional SARS-CoV-2 rRT-PCR results.

Jorge Cabrera Alvargonzález(a,b,c), Ana Larrañaga Janeiro(d), Sonia Pérez Castro(a,b,c), Javier Martínez Torres(e), Lucía Martínez Lamas(a,b), Carlos Daviña Nuñez (a), Víctor Del Campo-Pérez(f), Silvia Suarez Luque (g), Benito Regueiro García(a,b,g), Jacobo Porteiro Fresco(d)(*)

(a) Microbiology and Infectology Research Group, Galicia sur Health Research Institute (IIS Galicia Sur), SERGAS-UVIGO, Vigo, Spain

(b) Microbiology Department, Complexo Hospitalario Universitario de Vigo (CHUVI), Sergas, Vigo, Spain

(c) Universidade de Vigo, Vigo, Spain

(d) CINTECX, Universidade de Vigo, GTE, 36310 Vigo, Spain

(e) Applied Mathematics I, Telecommunications Engineering School, Universidad de Vigo, 36310 Vigo, Spain

(f) Department of Preventive Medicine and Public Health, Álvaro Cunqueiro Hospital, Vigo, Pontevedra, Spain

(g) Dirección Xeral de Saúde Pública, Consellería de Sanidade, Xunta de Galicia, Santiago de Compostela, A Coruña, Spain

(h) Microbiology and Parasitology Department. Medicine and Odontology, Universidade de Santiago, Santiago de Compostela, Spain

(*) porteiro@uvigo.es +34 986 818799

SUPPLEMENTARY MATERIAL 1

This section of the supplementary material includes a brief description of the mathematical algorithms used for sample classification: Support Vector Machines (i.e., SM) and Neural Networks (i.e., NN).

1. Support Vector Machine (SVM)

From the diverse existing classification algorithms, the first option tested was a support vector machine (SVM), a kernel-based network that performs linear classification on vectors transformed to a higher dimensional space, i.e., it separates these vectors by means of an optimal hyperplane in the feature space that contains the main characteristics of the baseline data. According to the kernel trick, the creation of such a feature space is obtained by the transformation $\phi: X \subset R^d \to U \subset R^s$, where $s \geq d$ (d represents the dimension of the original space, and s corresponds to the dimension of the feature space), allowing linear separating hyperplanes in the feature space to be equivalent to nonlinear separators in the original space.

SVMs tailored for classification are based on a sample $z^n, z_i = (x_i, y_i)$, where $x_i \in X \subset R^d$, $y_i \in Y = \{-1,1\}$, and $i = 1{:}n$ is linearly separable. I.e., it can be divided by a decision function $f_{w,b}(x) = sign(\langle w, x \rangle + b)$, where $w \in R^d$ and $b \in R$. The optimal separating hyperplane is defined as the maximum margin separator hyperplane that maximizes its distance to the classes. It has two major weaknesses: the requirement of linear separability of the sample and their linear character.

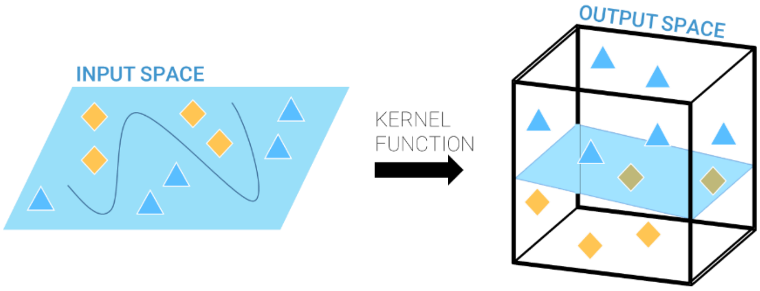

Supplementary figure 1: Support vector machine explanation.

In turn, this margin, or geometric margin, $\tau_{w,b}$, with respect to the sample z^n is described as

$$\gamma(\tau_{w,b}) = \min_{i\in\{1:n\}} \frac{1}{\|w\|} |\langle w, x_i \rangle + b| = \frac{1}{\|w\|} \min_{i\in\{1:n\}} |\langle w, x_i \rangle + b|$$

The optimal hyperplane is obtained as the solution of the following problem with its corresponding restrictions:

$$\max_{w\in R^d, b\in R} \left\{ \gamma(\tau_{w,b}) = \min_{i\in\{1:n\}} |\langle w, x_i \rangle + b| \right\}$$

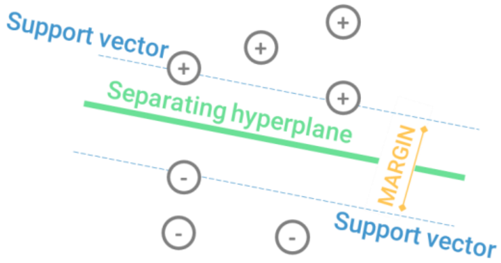

Supplementary figure 2: Maximum margin for a support vector machine.

Considering that this is a multiclass problem since more than two input parameters are considered for training, it is important to differentiate two approaches: *one-vs.-all* and *one-vs.-one*.

- One-vs.-All: This approach is based on building c SVM models where the ith classifier is trained with all the examples of the ith class coded as +1 and the

rest of the observations are coded ash -1 (hence its name one-against-all as each class is pitted against the rest).

- <u>One-vs.-One:</u> This methodology is based on the construction of *c(c - 1)/2* binary classifiers trained with elements of two classes 2 to 2. Thus, for each element of class i, the classification between the other elements of different classes can be established.

In the present work, both alternatives were tested. Since the SVM model used was optimized, it provided a better result in the one-versus-one application.

2. Neural Networks

Neural networks were born from the field of biology in the search to create a system that would follow the learning patterns of the human brain. This was achieved through the work of psychiatrist McCulloch and mathematician Pitts, whose first model consisted of an input layer (containing the original data); an output layer (containing the classification result) and a certain number of hidden layers with their corresponding number of nodes connected by weights, which were assigned based on common characteristics. Mathematically, a neural network can be denoted by the function $f: X \subset R^d \to Y \subset R^c$ [27] that can be expressed as follows:

$$f(x) = \phi(\psi(x)) \; \phi: X \subset R^d \to T \subset R^p \; \psi: T \subset R^p \to Y \subset R^c$$

where d is the dimension of the input space, p is the number of neurons of the hidden layer, c is the dimension of the output layer, T is the hidden space, ϕ is the activation function of the hidden layer and ψ is the activation function of the input layer.

For the case of artificial neural networks called *multilayer perceptrons* (MLPs) that are characterized by having a series of neurons called *perceptrons*, a back propagation

process that propagates the error back to the training in order to reduce the error until the NN learns the necessary information is used. Its expression can be shown as:

$$f(x) = \sum_{j=1}^{p} \phi_j(c_j \psi\left(w_j^T x + w_0\right) + c_0$$

where w_j and w_0 are the weights of the input layer and c_j and c_0 are the weights of the hidden layer.

In this case, the neural network utilized was a feedforward, fully connected model specialized for classification (Supplementary figure 3). This means that all the neurons in each layer are connected to the neurons in the previous layer, with each fully connected layer multiplied by a weight matrix plus a bias vector that must be considered. Moreover, an activation function, which was previously mentioned, is placed between the layers, allowing nonlinear learning of patterns between layers. Finally, the last layer followed by the softmax activation function results in the network soluti[illegible]

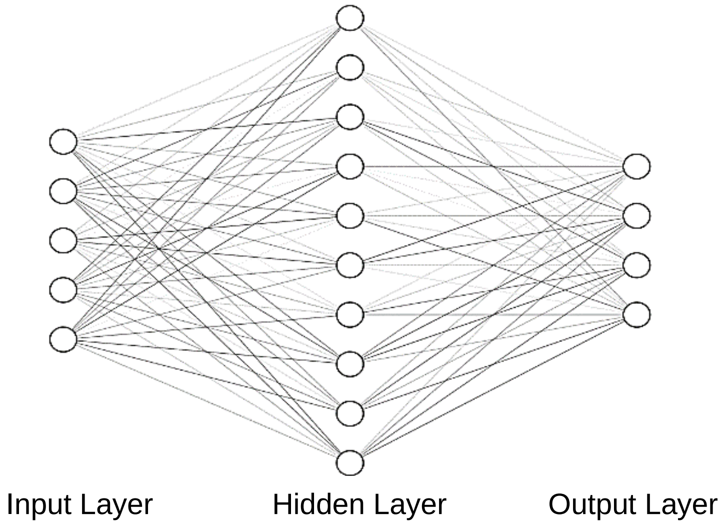

Supplementary figure 3: Feedforward, fully connected neural network structure.
